# Identifying related cancer types based on their incidence among people with multiple cancers

**DOI:** 10.1186/1742-7622-3-17

**Published:** 2006-11-08

**Authors:** Chris D Bajdik, Zenaida U Abanto, John J Spinelli, Angela Brooks-Wilson, Richard P Gallagher

**Affiliations:** 1 Cancer Control Research Program, BC Cancer Research Centre, 675 West 10^th ^Ave, Vancouver BC, Canada V5Z 1L3; 2 Department of Health Care & Epidemiology, University of British Columbia, Vancouver BC, Canada; 3 Canada's Michael Smith Genome Science Centre, BC Cancer Research Centre, 675 West 10^th ^Ave, Vancouver BC, Canada V5Z 1L3

## Abstract

**Background:**

There are several reasons that someone might be diagnosed with more than one primary cancer. The aim of this analysis was to determine combinations of cancer types that occur more often than expected. The expected values in previous analyses are based on age-and-gender-adjusted risks in the population. However, if cancer in people with multiple primaries is somehow different than cancer in people with a single primary, then the expected numbers should not be based on all diagnoses in the population.

**Methods:**

In people with two or more cancer types, the probability that a specific type is diagnosed was determined as the number of diagnoses for that cancer type divided by the total number of cancer diagnoses. If two types of cancer occur independently of one another, then the probability that someone will develop both cancers by chance is the product of the individual probabilities for each type. The expected number of people with both cancers is the number of people at risk multiplied by the separate probabilities for each cancer. We performed the analysis on records of cancer diagnoses in British Columbia, Canada between 1970 and 2004.

**Results:**

There were 28,159 people with records of multiple primary cancers between 1970 and 2004, including 1,492 people with between three and seven diagnoses. Among both men and women, the combinations of esophageal cancer with melanoma, and kidney cancer with oral cancer, are observed more than twice as often as expected.

**Conclusion:**

Our analysis suggests there are several pairs of primary cancers that might be related by a shared etiological factor. We think that our method is more appropriate than others when multiple diagnoses of primary cancer are unlikely to be the result of therapeutic or diagnostic procedures.

## Background

There are several reasons that someone might be diagnosed with cancer at more than one anatomic site. First, a new cancer might be caused by the therapy for a previous cancer. The risk of breast cancer is significantly increased among women who were treated for Hodgkin Disease with radiation [1]. Second, cancer might occur at multiple sites because a factor is associated with cancer at each site. Germline mutations in mismatch repair genes can produce susceptibility to cancers of the colorectum, ovary, stomach, small bowel, upper uroepithelial tract, hepatobiliary tract and brain [2]. Likewise, cigarette smoking affects the risk of several cancer types. Third, a different cancer type might be diagnosed because of diagnostic or surveillance procedures associated with a previous cancer. Cancers of the prostate are more likely to be diagnosed in men with bladder cancer because both can be diagnosed during the physical examination performed by a urologist. Finally, many people are diagnosed with more than one cancer because of chance. More than a third of Canadians is diagnosed with cancer during their lifetime [3].

Whatever the explanation, the non-chance incidence of multiple cancers in someone implies the diseases are related. Several previous analyses have compared the observed and expected incidence of all cancer types in people with multiple diagnoses [1,4-8]. The expected values in these analyses are based on age-and-gender-adjusted risks in the population. The time between diagnoses is usually considered an indication of the second cancer's cause. However, if cancer in people with multiple primaries is somehow different than cancer in people with a single primary, then the expected numbers should not be based on the risks among people with a single primary. A recent report recommended the design of new epidemiological methods to study second primary cancers [9]. We compare the observed and expected values of second primary cancers in British Columbia (BC), Canada, where the expected number for each cancer type ignores a person's age at diagnosis, and the time between cancer diagnoses. We think that our method is appropriate if an underlying risk factor, either genetic or environmental, is responsible for both cancer types.

The aim of this analysis was to determine what types of cancer occur more often than expected in people who are diagnosed with multiple primary cancers. We did this using records of cancer diagnoses between 1970 and 2004 from the BC Cancer Registry (BCCR). Permission to report the analysis was received from the Research Ethics Board at the University of British Columbia.

### Analysis

The data comprised records of all people who were diagnosed with more than one primary invasive cancer in BC between 1970 and 2004. Primary cancer refers to dysplastic growth that is not the metastases of another tumor. Invasive cancer is disease that is neither benign nor *in situ*. The data were obtained from the BCCR, a population-based registry that includes medical records for all cancer that have occurred in the population since 1962. The BCCR has a high level of data completeness based on standard international measures of cancer registration [10]. The diagnoses were classified as distinct cancer types according to the anatomic sites reported by the National Cancer Institute of Canada [3] and are defined using the International Classification of Disease for Oncology (ICD-O [11]). We excluded patient records that showed multiple diagnoses of the same cancer type because we were concerned these diagnoses might refer to the recurrence of previous disease.

A two-way table was created in which both the rows and columns represent cancer types. A table cell is the observed number of people (O) in the dataset with a diagnosis of both the types specified by the row and column. People who were diagnosed with more than two cancer types contributed to each relevant pair-wise tally. For example, someone diagnosed with each of lung, colorectal and stomach cancer would contribute 1 to the pairwise observations of lung-and-colorectal cancer, lung-and-stomach cancer, and colorectal-and-stomach cancer.

In people with two or more cancer types, the probability that a specific type is diagnosed can be determined as the number of diagnoses for that cancer type divided by the total number of cancer diagnoses. If two types of cancer occur independently, then the probability that someone will develop both cancers by chance is the product of the individual probabilities for each type. The expected number of people with both cancers (E) is the number of people at risk multiplied by the separate probabilities for each cancer. Explicitly, if there are N diagnoses of cancer in total, O_A _diagnoses of cancer type A and O_B _diagnoses of cancer type B, then

E_AB _= (O_A_/N) × (O_B_/N) × N     (1)

where E_AB _is the expected number of people who will be diagnosed with both cancer types A and B. The observed-expected ratio (O/E) is a measure of the cancer types' relatedness in the population. For people with more than one type of cancer, an O/E value of 1 indicates that a pair of cancer types was diagnosed as often as would be expected given the likelihood of being diagnosed with each cancer type. An O/E value less than 1 indicates a pair is less likely to be observed than all multiple primary records suggest, and an O/E value greater than 1 indicates a pair of cancer types occurs more frequently than would be expected. An approximate 95% confidence interval (95% CI) is O/E ± (1.96/√E), and we describe the relatedness of a cancer pair as "significant" when this confidence interval excludes 1.

## Results

**Figure 1:**
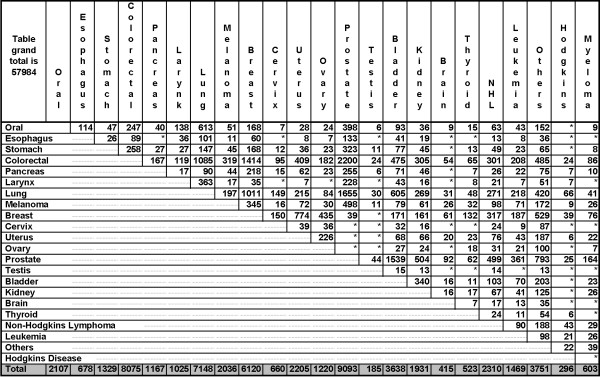
The number of cancer diagnoses in British Columbia among people with diagnoses of two or more types of primary cancer between 1970 and 2004. To ensure patient confidentiality in our population, all tables entries ≤5 have been replaced by an asterisk (i.e., *). Note that row totals (not shown) are the same as the corresponding column totals.

There were 28,159 people with records of multiple primary cancers in the BCCR between 1970 and 2004, including 1,492 people with between three and seven diagnoses. A cross-tabulation of the observed values for each pair of cancer types is shown in Figure 1. The most common pairs were prostate-colorectal, prostate-lung and prostate-bladder. These pairs were observed in males only, and reflect that each of the individual cancer types is relatively common. The next-most common pair was breast-colorectal and almost all paired diagnoses were observed in women. As in the three most-common pairs, the fourth pair reflects that each of the individual cancer types is relatively common in BC women. Together, the four most-common pairs reflect that cancer in BC is more common among men than among women. Approximately 6% of diagnoses in Table 1 were classified as "other" cancer and occurred at unspecified, ill-defined and unknown sites.

**Table 1:** Combinations of cancer types with two times or more observed cases than expected.

Pair of Cancer Types	Diagnosis of Both Cancers		O/E Ratio and 95%CI
		
	Observed (O)	Expected (E)	
**Women**			

Hodgkin Disease – Ovary	2	0.67	3.0 (0.6, 5.4)
Myeloma – Oral	2	0.69	2.9 (0.5, 5.3)
Bladder – Brain	2	0.80	2.5 (0.3, 4.7)
Esophagus – Melanoma	3	1.26	2.4 (0.7, 4.1)
Cervix – Oral	7	3.05	2.3 (1.2, 3.4)
Leukemia – Pancreas	5	2.30	2.2 (0.9, 3.5)
Brain – Oral	2	0.94	2.1 (0.1, 4.1)
Esophagus – Leukemia	2	0.95	2.1 (0.1, 4.1)
Larynx – Ovary	4	1.93	2.1 (0.7, 3.5)
Kidney – Oral	7	3.46	2.0 (0.9, 3.1)
			
**Men**			

Larynx – Leukemia	5	1.02	4.9 (3.0. 6.8)
Esophagus – Pancreas	2	0.44	4.6 (1.6, 7.6)
Bladder – Hodgkin Disease	4	1.03	3.9 (2.0, 5.8)
Bladder – Thyroid	6	2.37	2.5 (1.2, 3.8)
Kidney – Larynx	14	5.70	2.5 (1.7, 3.3)
Brain – Pancreas	2	0.87	2.3 (0.2, 4.4)
Larynx – Melanoma	14	6.55	2.1 (1.3, 2.9)
Kidney – Oral	29	13.90	2.1 (1.6, 2.6)
Esophagus – Melanoma	8	3.85	2.1 (1.1, 3.1)
Brain – Stomach	3	1.45	2.1 (0.5, 3.7)
Brain – Larynx	3	1.47	2.0 (0.4, 3.6)

For men and women separately, Table 2 shows the combinations of cancer types for which there were at least two people diagnosed with both cancers and there is more than twice as many observed cases as expected. Among women, only the combination of cervical with oral cancer has an observed O/E ratio for which the 95% confidence interval excludes 1. Among men, several combinations of cancer types have O/E ratios for which the 95% confidence interval excludes 1. Among both men and women, the combination of esophageal cancer with melanoma, and kidney cancer with oral cancer, are observed more than twice as often as expected. However, for neither combination in women does the ratio's 95% confidence interval exclude 1.

## Discussion

Our analysis suggests that several pairs of primary cancers might be related by a shared etiological factor. Someone's multiple primary cancer diagnoses instead might result from disease treatment or increased disease surveillance, but the lack of a specific temporal interval (or even order) between diagnoses inclines us to believe otherwise. The method of analysis that we propose in this paper is more appropriate than others when a shared etiologic factor is likely. An example of potential insights that are offered by the method is provided by multiple cancer diagnoses where one diagnosis is melanoma. Previous reports suggest that melanoma diagnoses occur more often than expected after diagnoses of lip cancer, prostate cancer, ovarian cancer, lymphoma and leukemia [7,12,13]. In addition, previous reports suggest that oropharyngeal cancer, brain cancer, prostate cancer, kidney cancer, lymphoma and leukemia are significantly more common than expected in patients with a prior diagnosis of melanoma [7,13-18]. Our analyses indicate that, in BC men, a diagnosis of melanoma either follows or preceeds a diagnosis of bladder, esophageal, laryngeal or lung cancer – and this happens significantly more often than expected. It is quite possible that factors, genetic or otherwise, affecting a man's risk of melanoma also affects his risk of bladder, esophageal, laryngeal and lung cancer. Likewise, risk factors for the other cancers might affect a man's melanoma risk.

We recently developed the computer software CGMIM [19] that reviews Online Mendelian Inheritance in Man (OMIM [20]) to identify genetically-related cancers [21]. OMIM is a computerized database of information about genes and heritable traits in human populations. CGMIM creates a matrix of observed/expected values as in Figure 1, but the values are determined by text-mining the gene descriptions in OMIM. Results are posted on the CGMIM website [19] monthly and suggest cancer types that are related genetically. Current CGMIM results (website accessed Sept 5, 2006) suggest that cancer type combinations involving the esophagus-pancreas, bladder-thyroid, brain-stomach and brain-pancreas are genetically related, but not other combinations reported in Table 1 of our analysis using BCCR data.

The relatedness of cancer types in this analysis might be the result of chance, but the large number of observations contributing to the analysis makes this unlikely. The absolute number of people with some pair of cancer diagnoses is not indicative of the relatedness of those cancers, because the probability of developing any pair of cancers depends on the probability of developing each cancer independently. A relatedness of cancer types must be based on their co-occurrence in excess of expectation. The expected number of people that are diagnosed with a pair of cancers is determined by the probability that each cancer is diagnosed individually. In general, the risk of a cancer depends on its incidence in the whole population – and not on the incidence in only people who have had two or more types of cancer. We calculated only the probability of diagnoses in people with more than one primary cancer, and the O/E value is a measure of the excess occurrence of two specific cancer types in people who have been diagnosed with multiple primary cancers.

Each of the cancer sites paired with melanoma in our analysis is distinct from the sites paired with melanoma in previous reports. Our results do not contradict earlier findings because the methods and assumptions that we use are different. In our example about cancers related to melanoma, current CGMIM results (website accessed Sept 5, 2006) indicate there are 16 genes that are related to both melanoma and bladder cancer. The same results indicate the gene ZNF202 is related to melanoma and cancers affecting the breast, cervix, ovary and lung. Recent reviews [22,23] suggests that several non-genetic risk factors that are associated with melanoma risk are also related to another type of cancer. For instance, occupational exposure to coal tar and pitches are risk factors for skin, lung and bladder cancer. Unlike previous analyses, we do not distinguish the order in which pairs of associated cancers occurred because there is no reason to assume the order isn't random. We also believe that someone's total number of diagnoses is likely to partly reflect sporadic events and not reflect the association between cancer types. An analysis that considers the order or total number of cancer diagnoses in someone might be useful, but the consequent reduction in analyzable observations would substantially limit the findings.

There are limitations that can affect the results from our method. It is possible that a type of cancer might go undiagnosed in some people, however cancer has serious health effects and undiagnosed cancer does not seem likely. Not all cancer diagnoses of BC residents might be recorded by the BCCR because of immigration or emigration, but we think that the likelihood of someone changing residences following a cancer diagnosis is small, particularly if it involves a change in healthcare. Healthcare services in BC are provided free to all residents. It is also possible that the method's results are affected by anatomic site definitions and cancer recording practices by the BCCR. However, the "expected" values in our method are calculated using "observed" totals, so the ratio of observed and expected values shouldn't be affected much.

Earlier suggestions for analyzing records from people with multiple cancer diagnoses have included: (1) eliminating diagnoses that are made within six months following an earlier diagnosis, (2) excluding diagnoses involving the same site or affected cell type, and (3) distinguishing cases by the time interval between diagnoses [24]. Those methods are intended to allow researchers to better interpret the results, but researchers should be careful not to be misled. Our analyses did not include cases of primary cancer that were diagnosed at the site of a previous invasive cancer, nor did we consider the time interval between cancer diagnoses. Our motivation was to distinguish cancer diagnoses that were unlikely to involve metastatic disease or a recurrence of the initial cancer. While new techniques improve the ability to distinguish recurrent and metastatic disease, the historical aspect of the data does not guarantee this problem did not occur in the past. Diagnoses that occur within six months may suggest that one cancer was detected because of clinical "work-up" resulting from the first diagnosis, but the cancers might be related by a shared factor. Likewise, two diagnoses involving the same cell type might indicate one cancer is the spread (i.e., metastasis) of another, but both cancers might be independent and yet etiologically related. Finally, the time between diagnoses of two cancers in someone might indicate whether the cancers are due to treatment, but it might also reflect the cancers' natural histories or the ages when they are most likely to present.

The etiology of most cancers is not fully understood, and the proposed method is meant to generate insight and new hypotheses. Additional criteria must be used to establish whether an etiologic factor affects two or more cancer types. Determining the relatedness of cancers might lead to new therapies because a treatment that is effective for one disease could be as effective for another. The relatedness of cancers is also important for providing targeted disease surveillance in all people who have diagnosed with cancer. Finally, the identification of related cancers is hoped to suggest a causal agent for some type of cancer because that same agent is known to affect the risk of a related type.

## Competing interests

The author(s) declare that they have no competing interests.

## Authors' contributions

All authors read and approved the final manuscript. JS, ABW and CB designed the data analysis. ZA and CB performed the data analysis.
